# Variations in Muscle Activity and Exerted Torque During Temporary Blood Flow Restriction in Healthy Individuals

**DOI:** 10.3389/fbioe.2021.557761

**Published:** 2021-03-19

**Authors:** Leonardo Gizzi, Utku Ş. Yavuz, Dominic Hillerkuss, Tommaso Geri, Elena Gneiting, Franziska Domeier, Syn Schmitt, Oliver Röhrle

**Affiliations:** ^1^Institute for Modelling and Simulation of Biomechanical Systems, Chair for Continuum Biomechanics and Mechanobiology, University of Stuttgart, Stuttgart, Germany; ^2^Department of Biomedical Signals and Systems, Faculty of Electrical Engineering, Mathematics and Computer Sciences, University of Twente, Enschede, Netherlands; ^3^Department of Neuroscience, Rehabilitation, Ophthalmology, Genetics, Maternal and Child Health (DINOGMI), University of Genova, Genova, Italy; ^4^Institute for Modelling and Simulation of Biomechanical Systems, Chair for Computational Biophysics and Biorobotics, University of Stuttgart, Stuttgart, Germany; ^5^Stuttgart Center for Simulation Technology (SC SimTech), University of Stuttgart, Stuttgart, Germany

**Keywords:** blood flow restriction, temporary ischaemia, HDEMG, motor control, somatosensory integration

## Abstract

Recent studies suggest that transitory blood flow restriction (BFR) may improve the outcomes of training from anatomical (hypertrophy) and neural control perspectives. Whilst the chronic consequences of BFR on local metabolism and tissue adaptation have been extensively investigated, its acute effects on motor control are not yet fully understood. In this study, we compared the neuromechanical effects of continuous BFR against non-restricted circulation (atmospheric pressure—AP), during isometric elbow flexions. BFR was achieved applying external pressure either between systolic and diastolic (lower pressure—LP) or 1.3 times the systolic pressure (higher pressure—HP). Three levels of torque (15, 30, and 50% of the maximal voluntary contraction—MVC) were combined with the three levels of pressure for a total of 9 (randomized) test cases. Each condition was repeated 3 times. The protocol was administered to 12 healthy young adults. Neuromechanical measurements (torque and high-density electromyography—HDEMG) and reported discomfort were used to investigate the response of the central nervous system to BFR. The investigated variables were: root mean square (RMS), and area under the curve in the frequency domain—for the torque, and average RMS, median frequency and average muscle fibres conduction velocity—for the EMG. The discomfort caused by BFR was exacerbated by the level of torque and accumulated over time. The torque RMS value did not change across conditions and repetitions. Its spectral content, however, revealed a decrease in power at the tremor band (alpha-band, 5–15 Hz) which was enhanced by the level of pressure and the repetition number. The EMG amplitude showed no differences whilst the median frequency and the conduction velocity decreased over time and across trials, but only for the highest levels of torque and pressure. Taken together, our results show strong yet transitory effects of BFR that are compatible with a motor neuron pool inhibition caused by increased activity of type III and IV afferences, and a decreased activity of spindle afferents. We speculate that a compensation of the central drive may be necessary to maintain the mechanical output unchanged, despite disturbances in the afferent volley to the motor neuron pool.

## 1. Introduction

The term blood flow restriction (BFR) refers to the practice of (temporarily) decreasing or interrupting the blood flow by the application of tight bandages or wrapping devices (Abe et al., 2012). The lack of blood circulation has profound metabolic and neural consequences. They are often exploited for training purposes (Loenneke et al., 2012b) and represent an elegant model to study of human neuromechanics. BFR causes alterations in different afferent channels (Mauritz and Dietz, 1980), it can be used for investigating human somatosensory integration mechanisms in healthy individuals, by means of transitory and reversible perturbations.

BFR has been used to train strength and induce hypertrophy, showing that loads as low as 30% of the maximal 1-repetition maximum (1RM) weight (Abe et al., 2012; Loenneke et al., 2012a; Yanagisawa and Sanomura, 2017), were as effective as 70% 1RM loads without BFR. While maintaining the training effect, blood flow restriction also showed reduced signs of exercise-induced tissue damage (Thiebaud et al., 2013). In force of its high effectiveness and general safety, BFR has been used to train cohorts of healthy individuals and stroke survivors (Murphy et al., 2019), as well as elderly populations suffering from muscle atrophy (Hughes et al., 2017). The chronic effects on force and muscle hypertophy of BFR-based training are relatively well-understood, as a conspicuous body of literature is present. In brief, it has been reported that BFR causes a quick depletion and a slow replacement of the locally available energy resources (e.g., phosphocreatine) and oxygen, together with an increase of catabolites and H^+^ ions (Yanagisawa and Sanomura, 2017). Metabolic stress and cell swelling are among the most prominent growth factors during BFR exercise (Loenneke et al., 2012b). It has been shown that this can trigger an increase in muscle protein synthesis as early as 3 h after a single bout of exercise (Fujita et al., 2007; Fry et al., 2010; Gundermann et al., 2012).

Despite the abundance of studies on chronic effects of BFR, its acute neural consequences have received relatively little attention. Yasuda et al. (2013) and Yasuda et al. (2014) reported that BFR causes an immediate increase in muscular activation, and that the effects tend to cumulate with either continuous or intermittent restriction. Other authors (Manini and Clark, 2009; Loenneke et al., 2012b) reported that the alteration of the peripheral environment during BFR may cause type I muscle fibres to fatigue faster than in control conditions and that, with the aim of maintaining the mechanical output unaltered, type II muscle fibres are preferentially recruited. The latter aspect, however, was never explicitly tested during BFR. Other authors report that the reduced force production of the muscle fibres during BFR-induced hypoxic conditions, may be linked to an increase in type III and IV (Manini and Clark, 2009) and a decrease in type I and II (Grey et al., 2001) afferent volley.

High-density Electromyography (HDEMG) and torque measurements allow to investigate the electrical and mechanical activity of a given anatomical district. From EMG, it is possible to estimate the overall activity of the motor neuron (MN) pool of a muscle, both in the temporal (root mean square, RMS) and spectral domains, as well as infer the average muscle fibre conduction velocity (MFCV). The information contained in the spectrum of the EMG is traditionally represented by its mean or median value (MDF) (Merletti et al., 2004). An increase in RMS, MDF or MFCV is traditionally associated to an increase in recruitment and firing rate of the motor neurons. MFCV is specifically associated to the recruitment of larger, fast-twitch muscle fibres. A decrease in median frequency and conduction velocity is reported in case of peripheral fatigue (Merletti et al., 2004). On the other hand, the spectral components of the torque signal are associated with the effective neural drive, e.g., the δ-band, (<5 Hz, De Luca et al., 1982), and e.g., the α-band (5–15 Hz, Halliday and Redfearn, 1958; Lippold, 1970; Laine et al., 2013, 2014) to the somatosensory feedback to the muscle.

In this study, we evaluate the acute effects of (partial or complete) ischaemia on the neuromechanical output of young, healthy volunteers by determining the alterations in specific EMG features and torque output, caused by temporary BFR. In particular, classical multichannel surface EMG parameters will be sided by the analysis of the spectral components of the torque and by the estimation of the average muscle fibres conduction velocity.

## 2. Materials and Methods

### 2.1. Participants

Healthy men and women aged 18–45 years were recruited based on a self-reported general state of good health, with no history of orthopaedic, cardiovascular or neurological conditions. Exclusion criteria were: recent (i.e., less than 6 months) injuries in the upper limb, presence of neurological diseases, hypertension, capillary frailty, deep veins thrombosis, haemophilia, or any disease correlated with altered (i.e., either reduced or increased) blood coagulation or in presence of pregnancy or suspicion of. Subjects using antidepressant, blood thinning/thickening or pain drugs were also excluded. In total, 12 healthy young individuals (29.83±4.15 years, 171.83±7.23 cm, 68.83±8.19 kg) were admitted to the study after signing an informed consent form. All the procedures complied with the Declaration of Helsinki and were authorized by the local Ethical Committee. The detailed anthropometrics of the subjects participating in this study are reported in Table 1.

**Table 1:** Participants anthropometrics table.

**Subj ID**	**Sex**	**Age (yrs)**	**Weight (kg)**	**Height (cm)**	**Systolic pr. (mmHg)**	**Diastolic pr. (mmHg)**	**BMI (kg/m**^**2**^)
S001	M	29	70	180	118	70	21.6
S002	F	25	58	166	130	90	21.05
S003	M	34	62	167	120	78	22.23
S004	F	28	72	160	118	78	28.13
S005	M	30	80	185	120	70	23.37
S006	M	34	75	168	126	86	26.57
S007	F	24	58	167	110	80	20.80
S008	F	27	60	168	120	80	21.26
S009	M	29	68	178	122	70	21.46
S010	M	27	74	176	122	74	23.89
S011	F	33	67	170	105	65	23.18
S012	M	38	82	177	120	80	26.17
Average		29.83	68.83	171.83	119.25	76.75	23.31
St. dev.		4.15	8.19	7.23	6.54	7.25	2.34

*The values are reported for each participant and summarized as average and standard deviation. Columns: Subject identification code (Subj ID), sex (Male, Female), age (years), weight (kg), height (cm), systolic and Diastolic pressure (mmHg), body mass index (BMI, kg/m^2^)*.

### 2.2. Data Recording

#### 2.2.1. Recorded Variables

Blood pressure (systolic and diastolic), elbow torque, and high-density electromyographic activity from the biceps brachii were recorded. Further, ratings of discomfort were quantified via a numerical scale rating (NSR, Williamson and Hoggart, 2005) ranging between 1 and 10. The subjects were instructed to associate 1 with “no discomfort at all” and 10 with “the worst imaginable discomfort”; data was reported verbally and recorded for each trial and condition immediately after the end of the contraction and prior to the deflation of the cuff. Data were then manually transferred to comma-separated files and electronically stored for further analyses.

#### 2.2.2. Experimental Apparatus and Subject Positioning

**Figure 1:**
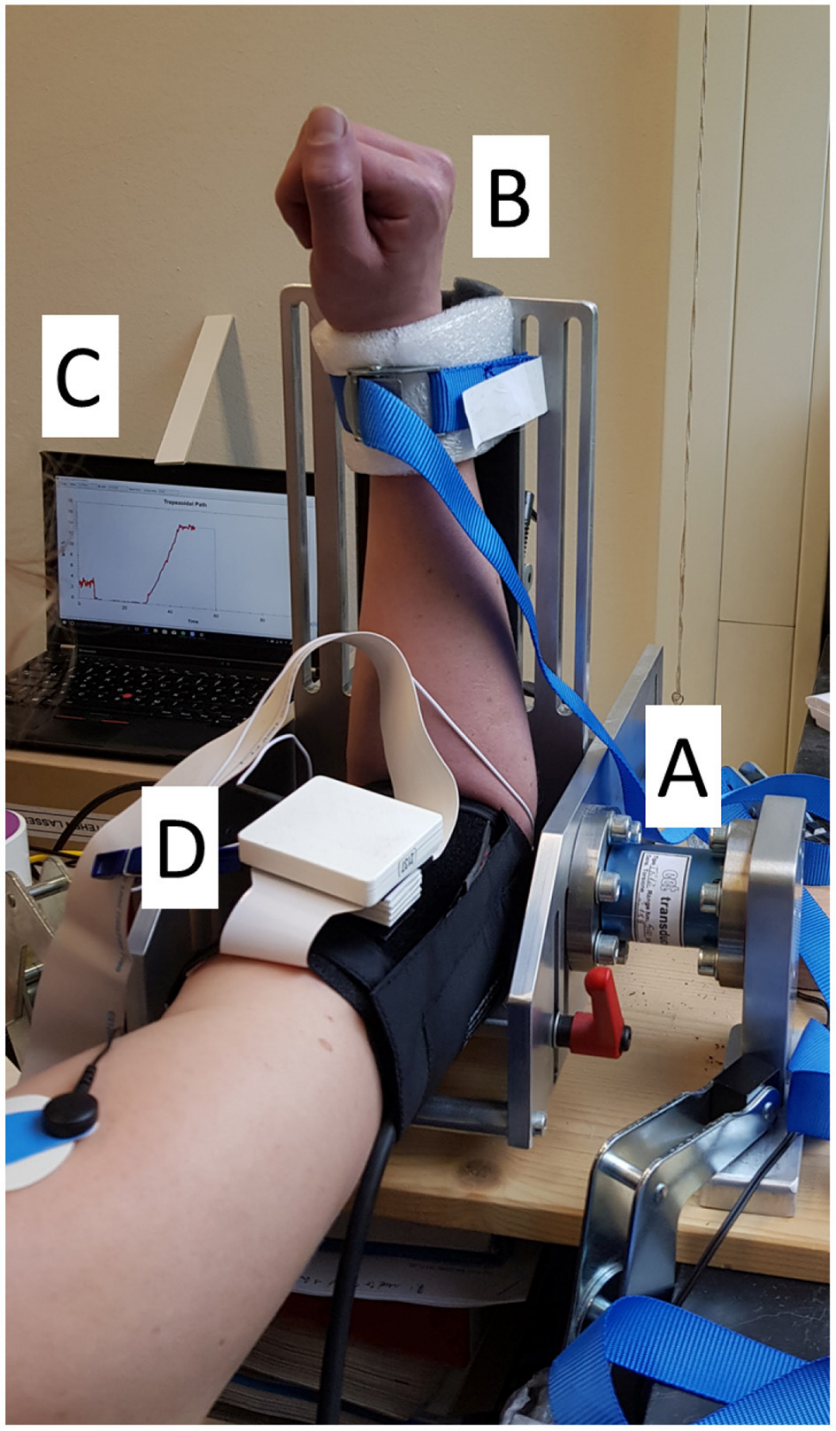
Experimental setup. The subject placed their right arm in the ALF apparatus **(A)** and were secured with an inextensible strap, softened by a foam cushion **(B)**. On the left it is possible to observe the visual feedback screen **(C)**. The HDEMG array was placed underneath the blood pressure cuff (only the pre-amplifier is visible in the picture, **(D)**.

Subjects were seated with a straight back on a chair and were instructed not to touch the back rest. Their right arm was placed in a custom-made aluminium rig (ALF, IMSB, University of Stuttgart, Stuttgart, Germany) whose sensing element was a 500 Nm flange-to-flange torque sensor (TR12, cct transducers, Turin, Italy). The height of the chair was adjusted so that a 90° angle for the elbow and for the abduction and flexion of the shoulder were obtained (see Figure 1). Finally, for the duration of the task, the wrist was restrained with inextensible belts (separated from the skin by a small foam cushion for comfort) to maintain it between pronation and supination. The length of the lever arm was noted down and checked for consistency at the beginning of each task. After the subject was positioned and restrained and prior to the beginning of each recording, a gravity compensation was performed by resetting the offset of the force amplifier.

#### 2.2.3. Recording Hardware and Software

Torque data was amplified (FORZA, OTBioelettronica, Turin, Italy; gain : 1,000 V/V). Torque and electromygraphic data were recorded by means of an EMG amplifier (QUATTROCENTO, OTBioelettronica, Turin, Italy; gain: 150 V/V, A/D depth: 16 bits per sample, sampling frequency: 2,048 samples per second, analog inputs: DC coupled, EMG: band-pass filter: 10–900 Hz, 8^th^-order Bessel filter) (Hermens et al., 2000). Data were recorded via a proprietary recording software (OTBiolab V.2.06, OTBioelettronica, Turin, Italy) and post-processed via custom written software in Matlab (2016b, the Mathworks, Natick, Massachusetts). Normalized torque values were displayed to the subject (visual feedback) on a computer screen (13 inches, 60 Hz refresh rate) which was placed roughly 1.2 m away from the subjects, in their line of sight.

#### 2.2.4. Subject Preparation

Subjects' skin was gently abraded (Everi Skin Preparation Gel, Spes Medica, Genova, Italy) and cleaned with alcohol (Kodan Tinktur forte, Schülke & Mayr, Norderstedt, Germany). A 64-channels square-shaped EMG matrix (ELSCH064NM3, OTBioelettronica, Turin, Italy) with 10mm inter-electrode distance was placed such that the center of the array would fall approximately at 1/3 of the distance between the acromion and the fossa cubit. The rows of channels were aligned with the estimated direction of the muscle fibres, following SENIAM recommendations (Hermens et al., 2000). The array was fixed with surgical tape (Fixomull Stretch, BSN Medical GmbH, Hamburg, Germany). Prior to placement, the holes of the array foam were filled with dense electro-conductive gel (AC CREAM250V-3, Spes Medica, Genova, Italy). Reference electrodes were placed on the medial epicondyle of the right elbow (local reference for the EMG preamplifier) and on the right acromion (reference for main amplifier). HDEMG data was recorded in referenced monopolar mode.

### 2.3. Experimental Tasks

#### 2.3.1. Experimental Protocol

Prior to the beginning of the measurements, the subjects were instructed about the scopes and procedures of the experiment. After a first blood pressure measurement the scientists prepared the subject for HDEMG and adjusted the apparatus. At this point a second blood pressure measurement was taken, the values retained, and the LP and HP levels computed. After a visual assessment of EMG signal quality, the subjects were given a few minutes to familiarize with the experimental apparatus and the visual feedback, then the measurements started.

#### 2.3.2. Blood Pressure Measurement

The blood pressure measurement was performed via an analog sphygmomanometer and cuff (boso classic, Bosch + Sohn, Jungingen, Germany) and a stethoscope (bososcope cardio, Bosch + Sohn, Jungingen, Germany) by an experienced operator. The measurement was always performed by the same researcher for one subject and was repeated twice: the first time after the signature of the informed consent, the second prior to the beginning of the tasks. During the blood pressure measurement, the subjects sat on the experimental chair, with their arm in the same position as in the experiment. The first measurement served the purpose of familiarizing the subjects with the blood pressure measurement procedure, and to avoid “white coat syndrome” phenomena (Pickering et al., 1988). The second measurement was used to determine the levels of pressure for the experiment.

#### 2.3.3. Torque Measurement

The maximal voluntary contraction (MVC) was recorded three times; repetitions were separated by at least 3 min, when the subjects were allowed to rest. During this time, they were disengaged from the experimental apparatus. Subjects were requested to reach their maximal force in around 3 s and to maintain it for 2–4 s, under verbal encouragement. Out of the three trials the global maximum value of the low-pass filtered force (4^th^-order Butterworth filter, 30 Hz cut-off frequency) was retained and used for normalization.

#### 2.3.4. Experimental Conditions

Three levels of force and three levels of cuff pressure were investigated, the variables were randomly picked to define the 9 experimental test cases for the subject. The force levels investigated were 15, 30 and 50% of the MVC, and the pressure levels determined as: atmospheric (AP—cuff deflated and valve open), low (LP—the average value between systolic and diastolic pressure) and high pressure (HP—1.3 times the systolic pressure).

#### 2.3.5. Execution of the Experimental Protocol

**Figure 2:**
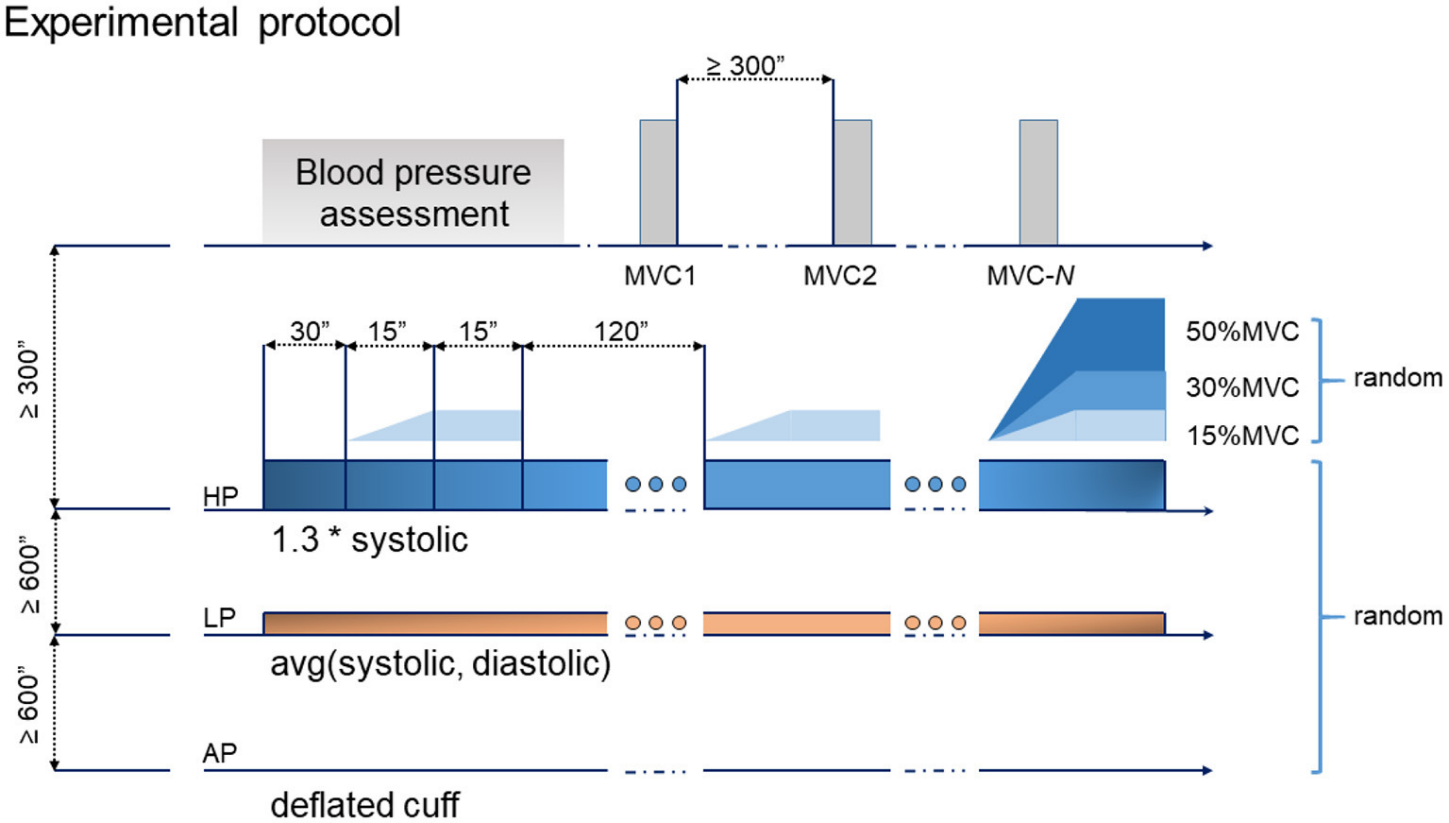
Experimental protocol: the tasks consisted of 3 repetitions of a ramp and hold exercise. Each ramp lasted for 15 s, followed by a 15 s hold. Three levels of pressure and torque were randomized leading to a total of 9 experimental test cases.

For each of the 9 investigated cases, the desired level of pressure was applied and maintained, then the recording started. During the AP condition the sphygmomanometer was inflated while the valve was left open in pretence, for blinding. After 30 s of baseline, the subjects flexed their elbow with the prescribed level of torque while following a straight ramp (lasting 15 s) being presented on the visual feedback monitor. After the ramp, they were asked to hold the same torque for another 15 s. Then, they rested for 120 s with the cuff inflated. The contraction was repeated three times (reported, from now on, as “trials”—T1, T2, and T3). At the end of the hold phase, the subjects were asked to report their level of discomfort. Throughout the resting phases, the external pressure was constantly adjusted to maintain the desired level. After the end of T3, the cuff was deflated and the subjects released from the constrains. Each condition was separated by a resting period of 10 min or more, to wash out the effects of BFR. During the recovery phase, the arm of the subjects was released from the constrains, and the subjects allowed to rest, while seated. The experimental sequence is summarized in Figure 2.

### 2.4. Data Analysis

#### 2.4.1. Data Segmentation

The central 13 seconds of the steady-state contraction were considered for further analysis. The torque and EMG data were processed in non-overlapping windows of 512 samples (250ms). Where appropriate, for each window one value per variable was computed.

#### 2.4.2. Torque Data Processing

Torque data was low-pass filtered (10^th^-order Butterworth filter, with a 3 dB frequency of 30 Hz) and normalized with respect to the MVC value. The coefficient of variation (CoV) was computed to estimate the variability in the stationary segment of torque signal. The root mean square (RMS) of the torque was calculated for each window and stored for further analyses.

The low-pass filtered torque data was de-trended by removing its mean and transferred to the frequency domain, the latter being needed to carry out the spectral analyses. The p-Welch's power density spectrum (Welch, 1967) was estimated over non-overlapping windows. To improve spectral resolution, 20480 discrete Fourier transform points were obtained by zero-padding. Finally, a Hamming window of length 2048 (equal to the sampling frequency) was applied. The spectrum was estimated separately for each trial, condition (pressure and level of torque) and subject. A confidence interval of 95 percent was chosen (Rosenberg et al., 1989; Yavuz et al., 2015). The normalized power of the spectrum—calculated as power/sum(power)—was also computed.

Additionally, each spectrum was divided into two frequency bands (1–5 Hz, and 5–15 Hz, respectively). The first one (δ-band) is considered to reflect the common drive, the second (α-band) is associated with physiological tremor and somatosensory information (Myers et al., 2004; Dai et al., 2017). For both bands, the area under the curve (AUC_δ_ and AUC_α_) was calculated with a numerical integration using a trapezoidal method with a uniform spacing. Lastly, for a direct comparison, the difference of both bands was calculated for each condition as in Equation (1).

(1) $$\begin{array}{l} AUC=\int_{a}^{b}f(x)dx=\frac{b-a}{2N}\sum_{n=1}^{N}(f(x_{n})+f(x_{n+1})). \end{array}$$

Thereby, *a* and *b* are the limiting frequencies 1–5 and 5–15 Hz, respectively, and *N* is the number of discretization intervals (50 for the first, and 100 for the second band, respectively). The total AUC (AUC_t_) from 1 to 20 Hz was computed to estimate variability in torque within the frequency domain.

#### 2.4.3. EMG Data Processing

For each trial and each subject a visual inspection of the EMG signal was performed and (a) remarkable spikes (compared with the surrounding samples), (b) strong power-line interference, (c) remarkably lower signal-to-noise ratio compared to their neighbors, (d) considerable offset (e) anomalous spectrum or e) clipping were marked and excluded from the analysis. EMG Data was then band-pass filtered (4^th^ order Butterworth band-pass filter between 20 and 450 Hz, see Hermens et al., 2000) and a single differential was obtained along the muscle fibres. Finally, the RMS and median frequency (MDF) (see Merletti et al., 2004) values for each channel and one value (see below) of the average muscle fibres conduction velocity were computed. To estimate the average MFCV, double differential signals were obtained from single differential along the fibres, they were interpolated 16 times and, for each pair of signals the time delay value was estimated by means of a cross-correlation based algorithm (Farina and Merletti, 2004; Sbriccoli et al., 2009; Klotz et al., 2019). The final value of MFCV was chosen as the lowest value across those, whose normalized cross-correlation exceeded a threshold of 0.8. Torque and EMG RMS data were normalized with respect to the value obtained during MVC. MDF and MFCV data were not normalized.

To infer the adjustments of the central nervous system to the perturbation over time, each computed variable was fitted with a linear function whose intercept and slope were retained for further analysis (Falla et al., 2003). Finally, to test for changes in the variability of neuromechanical output, the coefficient of variation was calculated.

#### 2.4.4. Statistical Analysis

For each variable and condition, data from all the subjects were pooled together and Shapiro–Wilk tests were applied to test for normality. As data were not normally distributed, a non-parametric test was used. Data were summarized using mean and standard deviation.

The influence of condition on the dependent variables was analyzed separately for each torque level using a Kruskal–Wallis rank-sum test. *Post-hoc* comparisons were performed by means of Dunn's test, with a Benjamini–Hochberg correction (alpha level set to 0.05/2). Despite the repeated nature of acquisitions, we considered the independent variables as inter-subject factors since they put subjects in different conditions. In particular, the 3 levels of the variable “trial” were considered diverse as the desired pressure level was maintained across T1, T2, and T3. The results were expected to produce differences between trials for each pressure level.

## 3. Results

### 3.0.5. Subjects' Discomfort

**Figure 3:**
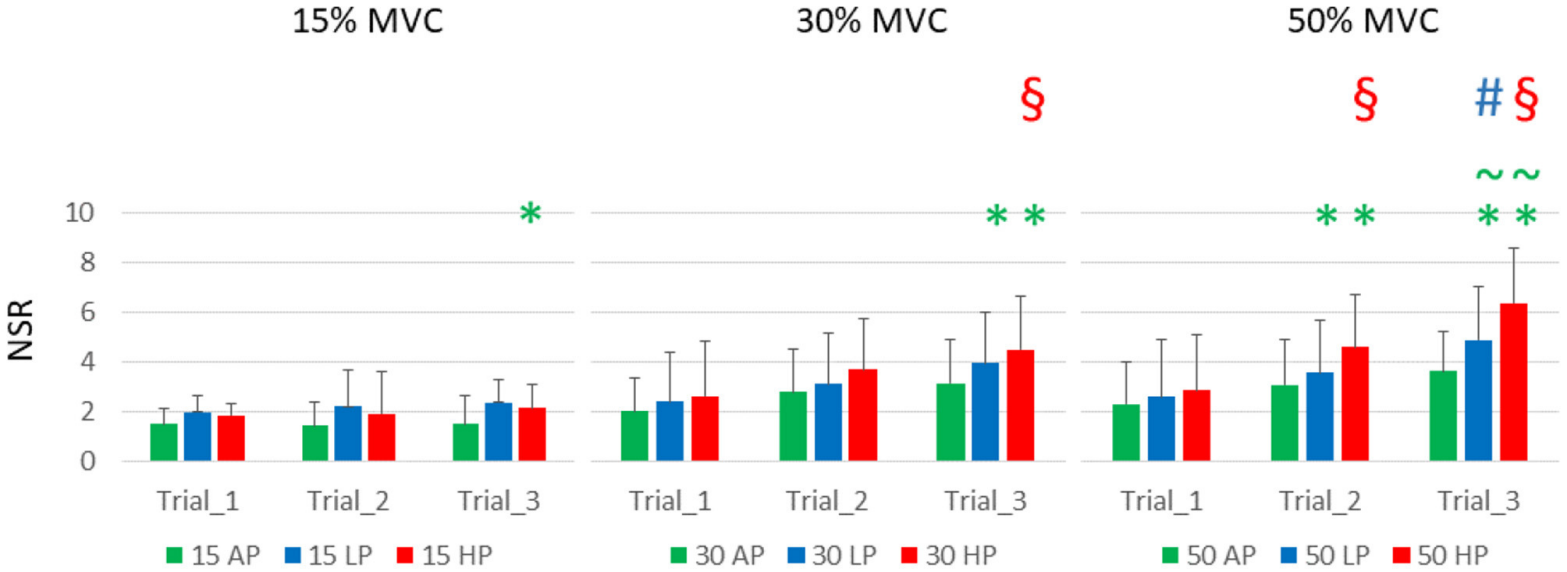
Subjects' reported discomfort. NSR is displayed as average and standard deviation across all the subjects for each condition and trial. It is possible to notice that the torque level had a relatively smaller effect on discomfort compared to the continuous application of blood flow restriction either partial (LP) or complete (HP). HP was responsible for the highest discomfort scores over time. It is important to notice that 4 subjects did not complete the last trial at 50%MVC HP, and they were asked to rate their discomfort at failure. Green asterisks (*) indicate statistical significance against AP1 for the same level of torque, green tilde (~) indicates significance against AP3, red section sign (§) against HP1, and the blue hash sign (#) against LP1 for and alpha level of 0.025).

Subjects reported an increasing level of discomfort at increasing pressure levels, torque, and time. Discomfort ranged, on average, from 1.5 ± 0.67 to 6.38 ± 2.2 (for 15% MVC at AP1, and 50% MVC at HP3, respectively); the results are detailed in Figure 3 and Table 2.

**Table 2:** Reported discomfort.

**NSR**			
**Condition**	**Trial 1**	**Trial 2**	**Trial 3**
AP 15% MVC	1.5 ± 0.67	1.46 ± 0.66	1.5 ± 0.48
AP 30% MVC	1.98 ± 0.97	2.23 ± 1.48	2.37 ± 1.71
AP 50% MVC	1.88 ± 1.17	1.93 ± 0.91	2.14 ± 0.94
LP 15% MVC	2.04 ± 1.32	2.81 ± 2	3.15 ± 2.25
LP 30% MVC	2.42 ± 1.69	3.11 ± 2.04	3.96 ± 2.03
LP 50% MVC	2.63 ± 1.75	3.69 ± 2.05	4.5 ± 2.13
HP 15% MVC	2.31 ± 1.72	3.08 ± 2.3	3.64 ± 2.25
HP 30% MVC	2.62 ± 1.81	3.58 ± 2.1	4.91 ± 2.08
HP 50% MVC	2.87 ± 1.58	4.6 ± 2.11	6.38 ± 2.2

*Overview of the discomfort levels for different conditions. Data is represented as average and standard deviation of the reported Numeric Scale Rating (range 1–10). Rows, conditions; columns, trial number; AP, atmospheric pressure; LP, low pressure; HP, high pressure*.

The reported levels of discomfort at the end of T1, for the same level of torque, did not increase with external pressure levels. They also did not change significantly across different levels of torque, although the trend shows higher values for LP and HP, compared to AP (NSR at T1, 15% MVC: AP 1.5 ± 0.67, LP 2.04 ± 1.32, HP 2.3 ± 1.71; 30% MVC: AP 1.98 ± 0.97, LP 2.41 ± 1.68, HP 2.61 ± 1.81; 50% MVC: AP 1.87 ± 1.17 LP 2.63 ± 1.74, HP 3.03 ± 1.54).

Over time, the discomfort increased significantly with the interaction of torque and pressure level. At 15% MVC, the discomfort was significantly greater than the baseline only for HP3, which differed from both AP1 and AP3^1^. At 30% MVC, the effect was significant at T3 for both LP and HP^2^. At this level of torque, the NSR significantly increased between T1 and T3 at the highest level of pressure^3^. At 50% MVC, the discomfort was greater than the AP1 for both LP and HP, at both T2 and T3, but not at T1^4^. At T3, the difference was also significant between AP and both LP and HP^5^, and for T1 and T3 for both LP and HP^6^.

One subject reported dizziness and light-headedness after the third trial at 50% MVC HP, however, they could continue with the experiment. No subjects reported pain or discomfort in the days following the procedures two subjects reported the appearance of petechiae.

### 3.0.6. Torque Output

The level of torque increased linearly according to the prescribed values (average and standard deviations across all subjects, trials and pressure levels: 14.7 ± 1.2%; 29.9 ± 1.6%; 49.1 ± 1.8% MVC). It is worth noting that 4 subjects failed at completing the last trial at 50% MVC; nevertheless, they were able to complete the experiment after the recovery. Torque output did not show any significant change in the time domain across different levels of pressure and trials for slope, intercept or CoV for any given level of torque. The only exception was the CoV at 15% MVC HP3, which was significantly greater than AP2 and AP3^7^. For the same amount of torque, the CoV also increased between HP1 and HP3^8^.

**Figure 4:**
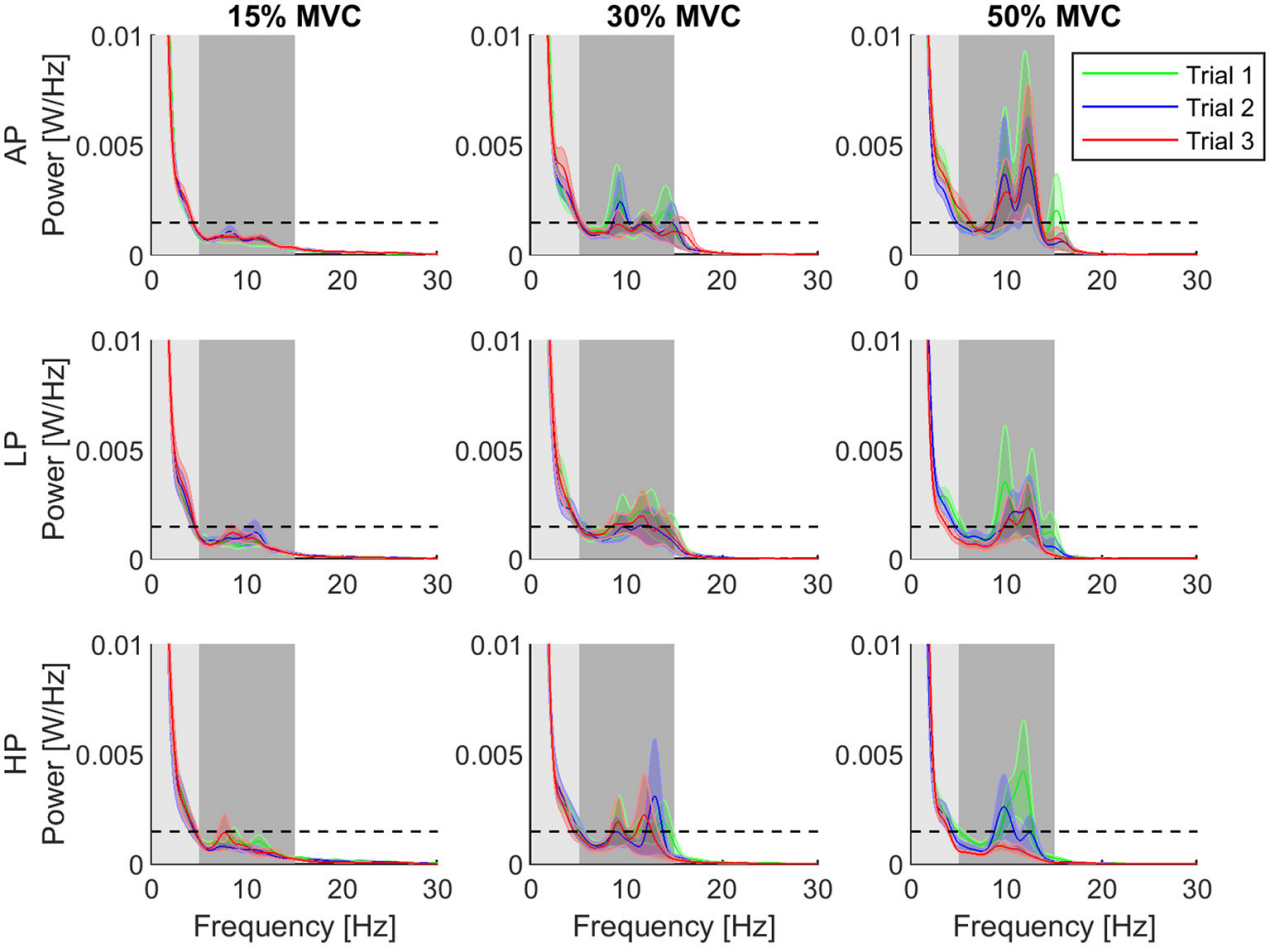
Normalized power spectral density for each condition. The third trial of the high pressure (HP) and torque level 50% is distinguishable decreased compared to the other conditions and trials. Furthermore, the error decreased clearly. Shaded areas showing the individual bandwidths from which the AUC_α_ (dark) and AUC_δ_ (light) were computed. The rows of the grid represent the pressure levels, whereas the columns are assigned to the different torque levels. The data is represented as average (solid line) and standard error (shade) across all the subjects for the three repetitions of the task. Trial 1 is represented in green, trial 2, in blue, and trial 3, in red. The dashed horizontal line depicts the 95% confidence level as a threshold indicating significance.

The power spectral density (PSD) of the torque at 15, 30, and 50% MVC, showed sensitivity to the level of external pressure over time (see Figure 4).

**Figure 5:**
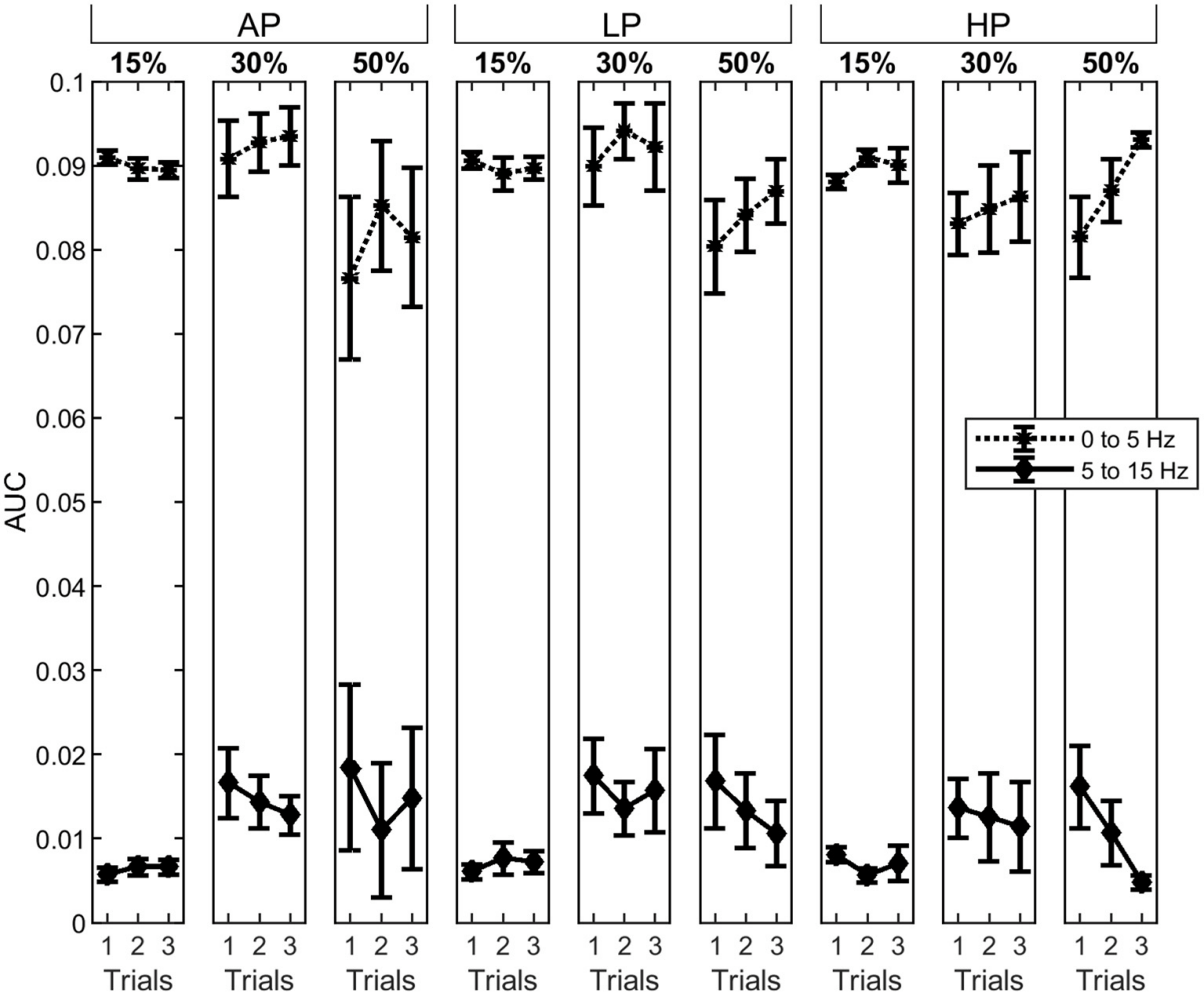
Area under the curve (AUC) the δ- (asterisks, dotted line) and α-band (diamonds, solid line). The columns represent the different torque levels. Within each level, the AUC for all three trials was calculated as the average with standard error. The power spectrum for the AUC has been normalized, previously. Whereas, the δ-band is decreasing for increasing torque levels, the α-band is increasing.

At 15% MVC, AUC_α_ was in general below the confidence level for all the conditions. The AUC_α_ and AUC_δ_ did not change through the trials at 15% and 30% MVC . At 50% MVC, they also did not change for AP and LP conditions . Despite a tendency of decreasing in AUC_α_ and increase AUC_δ_ values at 50% MVC, no significant differences were observed throughout the trials. When the AUC values were compared across conditions (AP, LP and HP), we found that the power in the α-band for T1 was significantly higher in HP than for AP at 15% MVC^9^. Furthermore, in the last trial (T3), AUC_α_ significantly decreased with increasing pressure at 50% MVC^10^. The power at δ-band, however, did not change under any conditions or trials. The mean AUC values and their standard error for each bandwidth are shown in Figure 5.

The AUC_t_ increased gradually through HP trials at 15 and 30% MVC^11^. Analysis between conditions showed that the total computed area from the last trial was significantly lower in AP than in LP and HP^12^ conditions for 50% MVC. The results can be interpreted on the basis of strong correlation between the total power density area underneath the α- and δ- bands and by the variation in torque.

### 3.0.7. EMG

**Figure 6:**
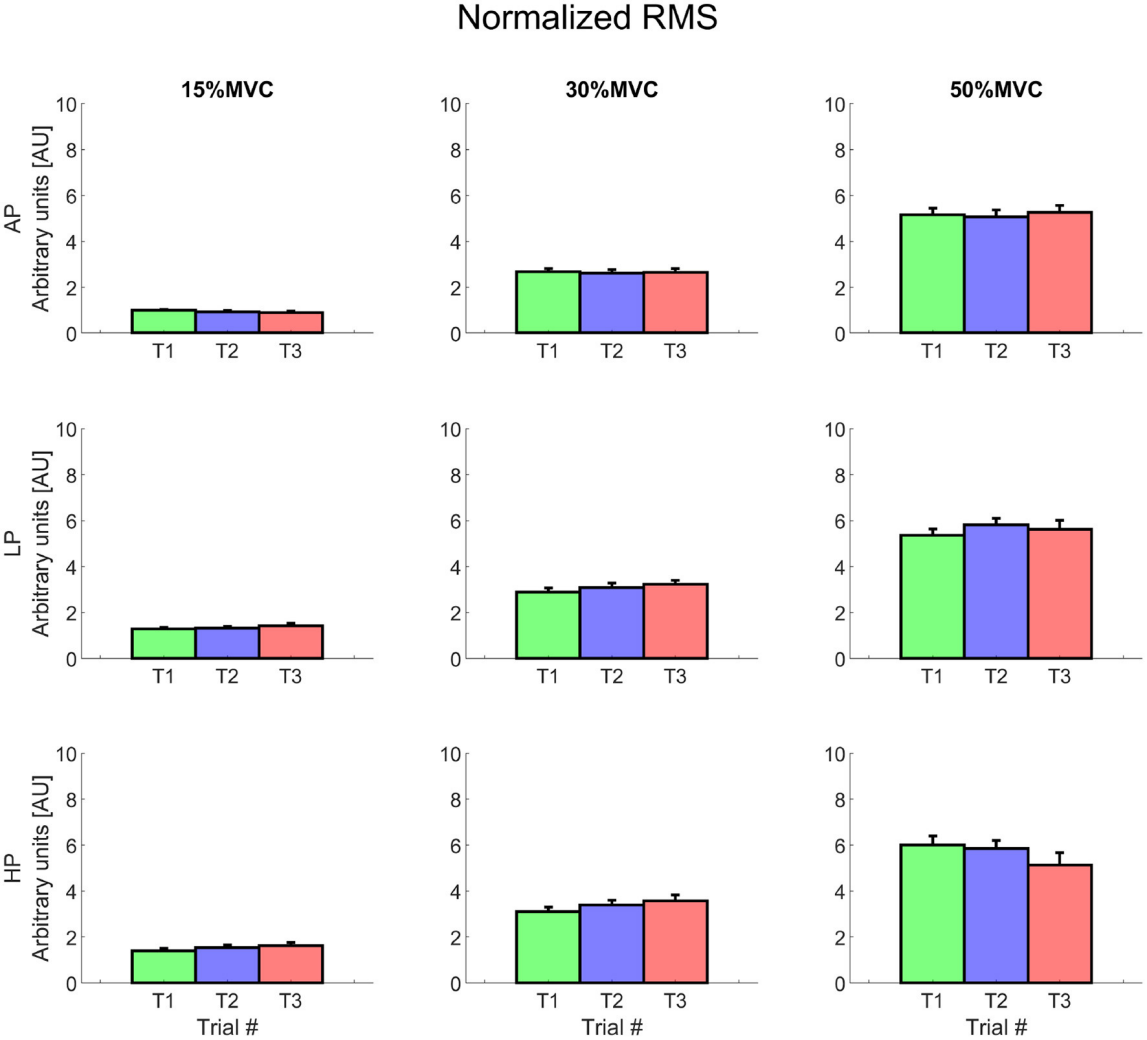
Normalized root mean square value for each condition. Rows represent different levels of pressure, columns, different levels of torque. The data is represented as average and standard error across all the subjects for the three repetitions of the task. Trial 1 is represented in green, trial 2, in blue, and trial 3, in red. Data is normalized with respect to the MVC value.

The energy associated to the EMG signal (RMS) increased over different levels of torque (about 2.5–3 times for 30% MVC and up to 6 times for 50% MVC) (Figure 6). The intercepts of the RMS showed significant differences at 15% MVC for both LP and HP, but did not change at the other levels of torque (AP 0.09 ± 0.03, LP 0.14 ± 0.04, HP 0.15 ± 0.04, average across T1, T2, and T3). At 15% MVC, all the AP levels were always lower than those at LP , except for AP1 (which only differed from LP3^13^). Similarly, RMS intercept values in AP2 and AP3 trials were lower then those at HP ; AP1 value at 15% MVC, however, was not significantly smaller than HP1, but only than HP2 and HP3^14^.

For the same level of torque, the RMS was relatively constant at 15 and 30% MVC and increased over time at 50% MVC (but only for AP). In particular the RMS showed a relatively more positive slope for AP, which was significantly greater than during HP for T2 and T3^15^.

**Figure 7:**
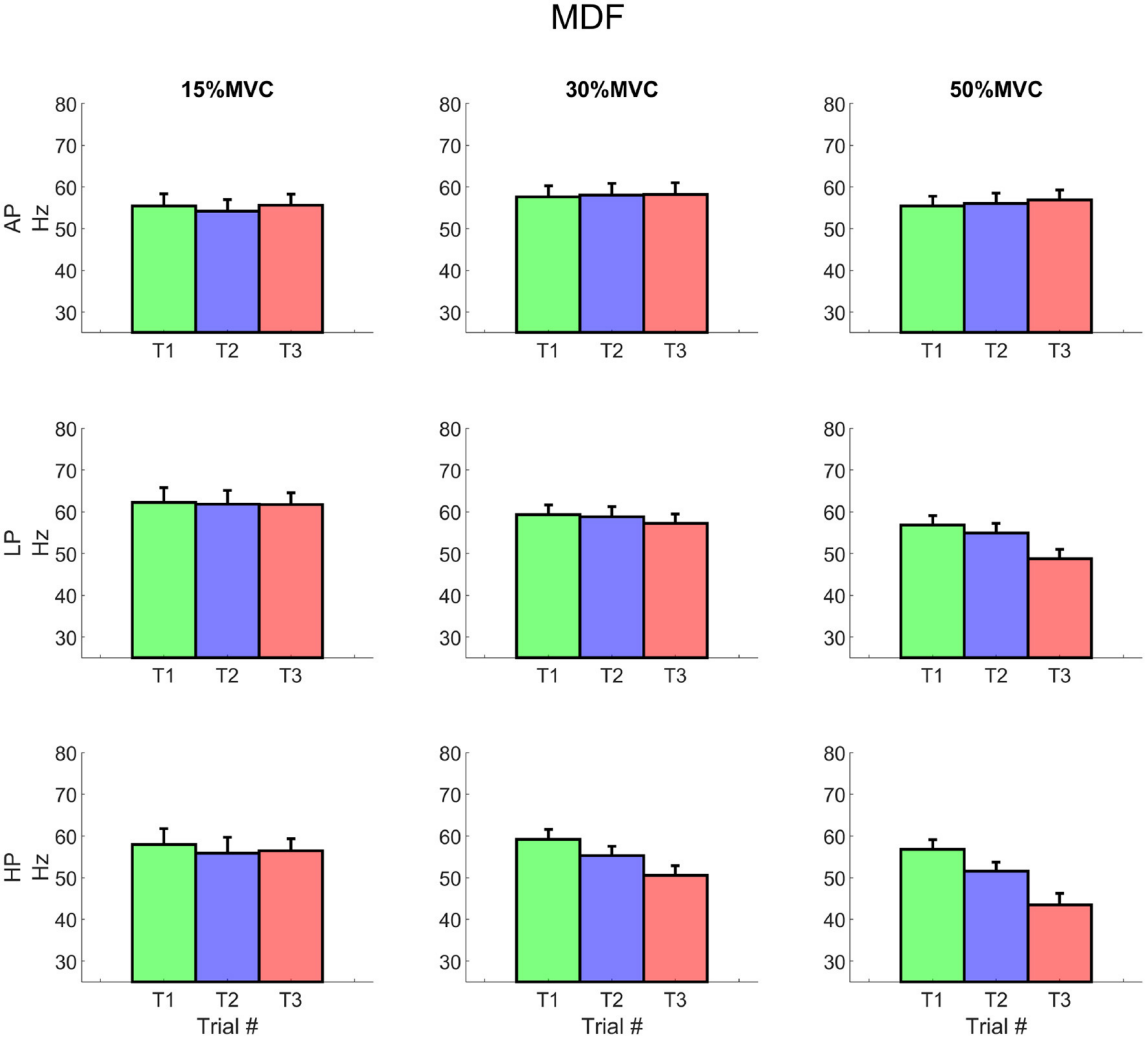
EMG median frequency for each condition. The values represent the average of all included channels across the matrix. It is worth noting the decrement of the variable at 30% MVC for LP3 and at 50% MVC for both LP and HP respectively, starting from Trial 2. Rows represent different levels of pressure, columns, different levels of torque. The data is represented as average and standard error across all the subjects for the three repetitions of the task. Trial 1 is represented in green, trial 2, in blue, and trial 3, in red.

For all the investigated conditions, except for 15% MVC HP, the median frequency globally showed a (non-significant) increment of about 10% at the beginning of T1. The slope was negative for all the conditions and its inclination was proportional to the exerted level of torque. The median frequency showed similar levels of decay over time for each torque level and independent of the external pressure (Figure 7). The MDF intercept, however, changed over time for the most demanding condition (at 50% MVC HP1 = 59.3 ± 6.65 Hz, HP3 = 44.3 ± 10.78 Hz^16^). At T3, it also differed significantly between AP and HP (AP3 = 59.73 ± 6.3 Hz^17^).

**Figure 8:**
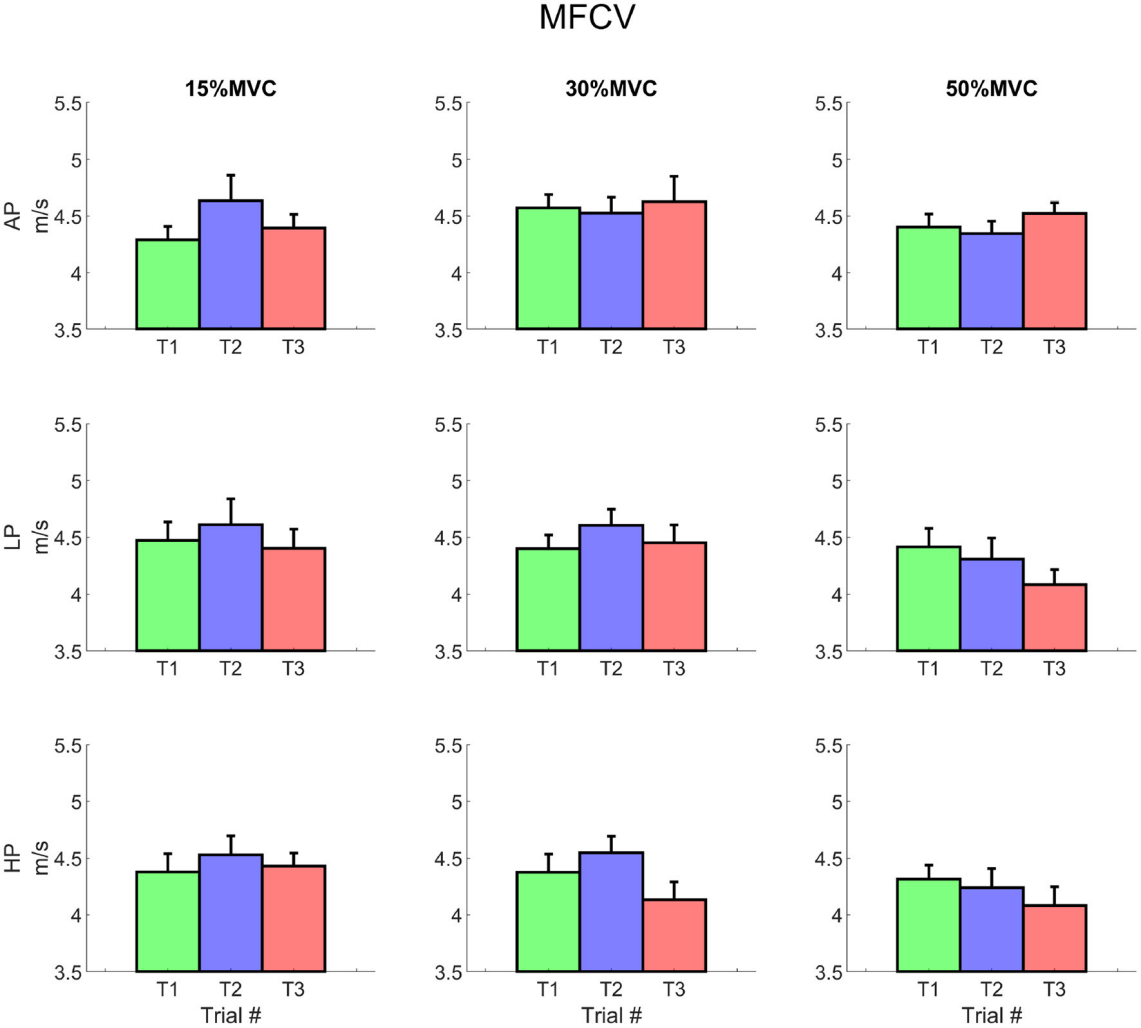
Average muscle fibres conduction velocity for each condition. It is possible to notice a decrease in the MFCV, which is most remarkable at trial 3 for 30 and 50% MVC for LP and for both LP and HP, respectively. Rows represent different levels of pressure, columns, different levels of torque. The data is represented as average and standard error across all the subjects for the three repetitions of the task. Trial 1 is represented in green, trial 2, in blue, and trial 3, in red.

The average MFCV did not change significantly over different levels of pressure and torque (Figure 8). At T1, the intercept value increased with torque for 15, 30, and 50% MVC (3.78 ± 1.49 m/s, 4.27 ± 1.12 and 4.41 ± 0.87 m/s, respectively; average across all pressure levels); values decreased—although not significantly—at T3 for 30 and 50% MVC (3.9 ± 1.47, 3.84 ± 1.52 m/s, respectively). The slope of the MFCV curve was negative and showed a trend inversely proportional to the exerted torque, but did not change over time (i.e., the MFCV showed always comparable slopes for T1 to T3).

## 4. Discussion

In this study we administered temporary blood flow restriction to 12 young healthy adults during isometric elbow flexions at different torque levels. To our knowledge, this was the first study investigating the immediate and time-dependent effects of BFR on neuromechanical variables, measured *during* the occlusion, by using HDEMG. This was also the first study investigating the BFR-induced changes in muscle fibres conduction velocity over time.

We report that despite the discomfort effect of BFR being exacerbated by the level of exerted torque and cumulated over a period of about 8 min, the majority of the subjects complete the entire protocol and none of them reported short or long term consequences. Torque amplitude parameters did not show changes, although spectral parameters did. The EMG analyses results are generally comparable across pressure conditions with expected differences across levels of torque. Taken together, the results show a strong fatiguing effect due to BFR, and allow for some interesting speculations about the mechanisms that the central nervous system uses to cope with the perturbation. In particular, our experiments suggest that a compensation of the central drive may be necessary to maintain the mechanical output unchanged despite disturbances in the afferent volley to the motor neuron pool.

For each level of torque, at T1 the reported discomfort and all the other parameters investigated were similar across all the conditions. This result suggests that the administered recovery time (>10 min) was sufficient to wash out the metabolic and neural effects of BFR perturbation. BFR is considered a highly-tolerable and quickly reversible exercise protocol (Husmann et al., 2018), which at the same time show remarkable immediate and long-term effects (Loenneke et al., 2015). The level of discomfort during partial or complete ischaemic block increased over time and its development was dramatically exacerbated at higher levels of exerted torque. This finding is compatible with an increase in hypoxia and acidosis and a depletion of available anaerobic substrates (e.g., phosphocreatine), an expected effect of BFR previously shown by the direct measurements of pH and lactate (see, for example Loenneke et al., 2012b; Yanagisawa and Sanomura, 2017).

Despite the discomfort, the required torque output was maintained in all the conditions by almost all the participants. Although the temporal parameters of torque did not change, during the most challenging conditions, their spectra showed a non-statistically significant reduction in the magnitude of the α- (5 Hz to 15Hz) and an increase in the magnitude of the δ- (<5Hz) bands. While the α-band is normally associated with physiological tremor oscillation and is considered to be highly dependent on proprioceptive sensory feedback (Halliday and Redfearn, 1958; Lippold, 1970; Laine et al., 2013, 2014), the δ-band is associated with the effective neural drive to the muscles (see De Luca et al., 1982). The results on the torque spectra allow a two-fold speculation: first, that the reduction in the α-band may be linked with an increase in type III and IV afferent volley, whilst type I and II may be decreased and, second, that the increase in the δ-band may reflect an increased overall descending drive to the motor neuron pool. An increment in type III and IV afference during BFR is supported by a large body of literature (e.g., Amann et al., 2009; Manini and Clark, 2009; Blain and Hureau, 2017) and reinforced by the discomfort sensation reported by our subjects. The role of the mechanosensitive type III and IV muscle afferents has been identified as a neural link between the CNS-mediated decrease in motoneuronal output and the degree of peripheral fatigue (Amann et al., 2009; Blain and Hureau, 2017). Similarly, a decreased type I (and especially type Ia) and II afferent volley is acknowledged during BFR (e.g., Grey et al., 2001; Christakos et al., 2006; Erimaki and Christakos, 2008) and anecdotally confirmed by our subjects (some of which reported a “decrease in the muscle tremor” after a few minutes). It should be noted, however, that although a complete ischaemia of over 15 min is considered to be necessary to fully block large axons afferents, it is reasonable to hypothesize that type I and II afferent volley may have already started decreasing earlier. The increase in the δ-band is ultimately suggestive of a necessity, for the central nervous system, to supply a stronger descending volley -driven by the visual feedback- to contrast the reduced excitability of the motor neuron pool caused by the ischaemic block. High variability of the low-frequency component suggests an inaccurate neural control of force due to pain and discomfort (Farina et al., 2012), most likely induced by long-time HP accommodation.

The median frequency of the EMG signal showed an increment at lower levels of torque for the higher pressure levels. For all the conditions, the slope of the MDF was negative and inversely proportional to the exerted torque. The decay of MDF did not change with different levels of pressure, but its intercept was lower over trials at HP. Spectral compression is traditionally considered as a manifestation of fatigue (Merletti et al., 2004) since it is linked to changes, at least, in the shape of the motor unit action potential (MUAP).

Whether the changes in the MDF are to be addressed entirely to peripheral effects of the muscle fibres fatigue, or if an influence of the motor neuron activity would also be present, cannot be discerned directly with the present study. Previous works on motor unit decomposition in similar settings (Hyngstrom et al., 2018; Fatela et al., 2019), however, agree on a reduction of the recruitment threshold and an increase in firing rate of individual motor units after the administration of BFR, suggesting that at least some effect on the motor neuron pool is present.

The energy associated to the EMG signal (RMS) increased at lower levels of torque for the higher pressure conditions, but did not increase for the higher levels of torque. This phenomenon was already observed in literature: some authors report that the increase in muscular activation is more prominent for low (down to 10–30%MVC) contraction levels and lower level of restriction (Takarada et al., 2000; Loenneke et al., 2015; Ishizaka et al., 2019), and others that, for higher levels of contraction, the EMG amplitude either remained the same (Dankel et al., 2018) or even decreased (Teixeira et al., 2018).

Finally, the muscle fibres conduction velocity showed signs of decay with slopes inversely proportional to the exerted torque and an intercept that decreased (although not significantly) at T3 for 30% MVC LP, and at 30 and 50% MVC at HP. This result is in line with the EMG spectral parameters during BFR exercises and is suggestive of fatigue phenomena.

The reported results over EMG activity (i.e., RMS, and MDF) are in line with previous studies, indicating an increase of the EMG amplitude for lower but not for higher levels of exerted force. A widely accepted explanation for this phenomenon is the preferential recruitment of type II muscle fibres when the lack of oxygen and the acidification of the muscle tissue prevents slow-twitch fibres from contracting (Manini and Clark, 2009; Loenneke et al., 2012b). Our study, however, challenges this explanation by directly estimating the changes in muscle fibre conduction velocity over time and conditions. A reduction of active type I muscle fibres in favor of type II, for the same amount of exerted torque, would have resulted in an overall increase of the MFCV or, at least, in a relatively less negative coefficient of its slope. The latter, instead, always showed comparable values across levels of external pressure and over time. This result is compatible with peripheral manifestations of fatigue, and is corroborated by the negative slope of the spectral parameters. This result taken together with the aforementioned motor units studies, suggests that the main contributor in the increase of the EMG amplitude may be the increase in the motor units firing rate.

An increase in the descending neural drive is likely to cause plastic effects whose longer-term consequence (an increased excitability of the MN pool) translates in the well-documented increase in the voluntary muscle force, as well as in the reported effects at the motor unit level in healthy individuals and stroke patients (e.g., Hyngstrom et al., 2018). In support of this hypothesis, Merletti et al. (1984) reported that the instantaneous median frequency showed signs of supercompensation after ischaemic blocks, for times varying between 20 and 40 min. Those results, taken together, foster the idea that an increase of type III and IV afferences (Manini and Clark, 2009), together with a reduction of type I and II, causes a general inhibition of the motor neuron pool excitability, that is centrally compensated. This phenomenon is believed to be correlated with the release of neuromodulators (e.g., serotonin, norepinephrine) (Sharma et al., 2015) that increase the overall excitability of the MN pool (Taylor et al., 2016) over time. This systemic effect could explain the benefit of BFR on muscle groups that are not reached by the ischaemic block and is compatible with the absence of changes in agonist-antagonist coactivation reported during temporary ischaemia (Sousa et al., 2017).

### 4.0.8. Limitations

It should be noted that it is not possible, with the current setup, to conclusively determine the proportion between peripheral (muscle fibre) and more central (motor neuron) effects of BFR on the neuromechanical output. Both EMG and torque outputs depend—to the very least- on the activity of the lower motor neurons of the spinal cord, and that of muscle fibres. In absence of a punctual description of the muscle anatomy and/or an at least partial decomposition of the motor output, the results should be considered carefully.

### 4.0.9. Outlook

Further measurements are necessary to determine (a) the effects of BFR on the MUAP shape (which could shed a light on the local changes to the muscle fibres during this type of perturbations) and (b) the changes it causes to the MN pool excitability, on the target limb and other anatomical districts. The first could be explained by either an increase in the excitability of the MN pool and/or by a chemically-mediated neuromodulation, the latter by the neuromodulation theory only. Those studies may explain the neural mechanisms linked to the adaptation that follows chronic exposure.

A structured and systematic approach to further investigate and test some of the hypotheses postulated herein are the use of detailed, biophysical neuromuscular models (Röhrle et al., 2019). For this purpose, existing models of the sensory, motor, and support structures (e.g., Heidlauf et al., 2016; Klotz et al., 2019; Schmid et al., 2019) need to be extended by blood perfusion (e.g., Koch et al., 2020) and metabolic models. However, once extended, they can be utilized with continuum-mechanical multi-muscle musculoskeletal system frameworks, (e.g., Röhrle et al., 2017; Valentin et al., 2018), to provide powerful ways to study the mechanisms of organ- and organism-wide somatosensory integration. This way, one can systematically investigate which of small-scale processes affect the musculoskeletal system in which way, i.e., testing the proposed hypotheses—something that is hardly possible in this detailed fashion employing experiments. Nevertheless, experiments are necessary for parameter estimation and validation.

Finally, although more rigorous measurements and simulations may be required, it is tempting to speculate that torque spectral analysis may represent an intriguing metric for an objective (i.e., independent on subject perception) measurement of discomfort.

## 5. Conclusions

BFR causes an immediate depression of the spinal MN pool excitability (likely caused by a decrease of type I and II, and an increase of type III and IV afferences), as a response to metabolic perturbation.

The results suggest that (a) an increase in the corticospinal (common) drive is necessary to reach similar levels of mechanical output during the administration of BFR and, as a consequence, (b) that the repeated exposure to BFR training may decrease the recruitment threshold of the whole MN pool, providing a neural facilitation for the production of force.

Conclusive proof of the effects of BFR on the motor neuron pool activity, however, still requires further investigation.

## Data Availability Statement

The raw data supporting the conclusions of this article are available from the authors upon reasonable request.

## Ethics Statement

The studies involving human participants were reviewed and approved by Ethical Committee of the University of Stuttgart, Approval number 18-003. The patients/participants provided their written informed consent to participate in this study.

## Author Contributions

LG, UY, and OR conceived the study. LG, DH, and EG performed the experiments. LG, UY, and DH analyzed the data. TG, UY, and LG performed the statistical analysis. All authors contributed in critically discussing the results of the study, and approved the final version of the manuscript.

## Conflict of Interest

The authors declare that the research was conducted in the absence of any commercial or financial relationships that could be construed as a potential conflict of interest.

## Footnotes

^1^(AP1 vs. HP3, *P* = 0.017; AP3 vs. HP3, *P* = 0.009).

^2^(AP1 vs. LP3, *P* = 0.019; AP1 vs. HP3, *P* = 0.005).

^3^(HP1 vs. HP3, *P* = 0.002).

^4^(AP1 vs. LP2, *P* = 0.018; AP1 vs. LP3; *P* = 0.002; AP1 vs. HP2, *P* = 0.001; AP1 vs. HP3, *P* < 0.001, respectively).

^5^(AP3 vs. LP3, *P* = 0.008; AP3 vs. HP3, *P* < 0.001).

^6^(LP1 vs. LP3, *P* = 0.002; HP1 vs. HP3, *P* = 0.002).

^7^(*P* = 0.008 and *P* = 0.004, respectively).

^8^(*P* = 0.006).

^9^(*P* = 0.017).

^10^(HP3-AP3: *P* = 0.047, LP3-AP3: *P* = 0.009).

^11^(at 15% MVC, T1-T2: *P* = 0.048, T1-T3: *P* = 0.009; at 30% MVC, T1-T3: *P* = 0.048).

^12^(*P* = 0.009 and *P* = 0.047, respectively).

^13^(*P* = 0.015).

^14^(AP1 vs. HP2, *P* = 0.017; AP1 vs. HP3, *P* = 0.007).

^15^(AP2 vs. HP2, *P* = 0.006, AP3 vs. HP2, *P* = 0.02).

^16^*P* = 0.022.

^17^*P* = 0.021.
